# Analyzing the Influence of Video Game and Music Engagement on Technical Skills Acquisition in Dental Students in Preclinical Phase: Protocol for a Prospective, Controlled, Longitudinal Study

**DOI:** 10.2196/55738

**Published:** 2024-09-13

**Authors:** Roselyne Clouet, Alexis Gaudin, Jeanne Tourtelier, Claire Gogendeau, Samuel Serisier, Tony Prud'homme

**Affiliations:** 1 Dentistry Department Nantes Hospital Nantes University Nantes France; 2 Université Polytechnique Hauts-de-France LARSH Valenciennes France; 3 Nantes Université, CHU Nantes INSERM, RMeS ( Regenerative Medicine and Skeleton) UMR 1229 Nantes France

**Keywords:** video game, music practice, dental education, technical skills, simulation, video games, music, preclinical skills, dental students, dental student, pre-clinical phases, longitudinal investigation, dentistry, gestures, gesture, preclinical training, training, protocol, tool, tools, cognitive skills, mobile phones

## Abstract

**Background:**

The practice of dental surgery requires a few different skills, including mental rotation of an object, precision of movement with good hand-eye coordination, and speed of technical movement. Learning these different skills begins during the preclinical phase of dental student training. Moreover, playing a musical instrument or video game seems to promote the early development of these skills. However, we found that studies specifically addressing this issue in the field of dental education are lacking.

**Objective:**

The main aims of this study are to evaluate whether the ability to mentally represent a volume in 3D, the precision of gestures with their right and left hand, or the speed of gesture execution is better at baseline or progresses faster for players (video games or music or both).

**Methods:**

A prospective monocentric controlled and longitudinal study will be conducted from September 2023 and will last until April 2025 in the Faculty of Dental Surgery of Nantes. Participants were students before starting their preclinical training. Different tests will be used such as Vandenberg and Kuse’s mental rotation test, the modified Precision Manual Dexterity (PMD), and performing a pulpotomy on a permanent tooth. This protocol was approved by the Ethics, Deontology, and Scientific Integrity Committee of Nantes University (institutional review board approval number IORG0011023).

**Results:**

A total of 86 second-year dental surgery students were enrolled to participate in the study in September 2023. They will take part in 4 iterations of the study, the last of which will take place in April 2025.

**Conclusions:**

Playing video games or a musical instrument or both could be a potential tool for initiating or facilitating the learning of certain technical skills in dental surgery.

**International Registered Report Identifier (IRRID):**

DERR1-10.2196/55738

## Introduction

### Background

The last 50 years have seen the emergence of playful practices such as video games, which are now an integral part of the digital cultural landscape. As a result, demographic changes have led to the emergence of a new generation of learners who live in a rich and accessible digital environment, surrounded by computers and smartphones. As a result, this generation is now more sensitive to and interested in the use of video games than previous generations seemed to be [1]. Today, video games have become one of the most popular leisure activities among young adults. Many studies are now focusing on the effects of this ludic practice, particularly in terms of learning. Some articles seem to suggest a link between the development of psychomotor and cognitive skills and the practice of video games [2]. Research dating back to the early 1980s has consistently shown that playing video games (regardless of genre) enables players to acquire faster reaction times, better hand-eye coordination, enhanced psychomotor skills, and improved self-esteem [3-5]. These elements have been found in various fields, including medical education. For example, a study using video games on the Nintendo Wii console showed bimanual improvement in surgery, including an increase in the precision of nondominant hand movements [6,7].

Video games may reduce the time required for medical students to acquire or improve surgical skills [1,8-10]. Some studies have also shown that previous video game experience improves baseline performance on a laparoscopic simulator and that student players have better visuospatial awareness when using the simulator [11,12]. Thus, playing video games would significantly reduce reaction time without compromising the precision of the technical gesture. An increase in execution speed has been observed in various tasks outside the gaming situation [8,13]. To date, no study seems to have evaluated the beneficial effects of video games on the preclinical training of dental students. However, precision, speed of execution, and 3D volume representation are skills that are required to perform dental treatment [14].

In addition, the first traces of musical practice date back to prehistoric times, approximately 35,000 years ago. Over the centuries, music has been listened to, played, and then analyzed in empirical studies that have demonstrated the benefits of playing a musical instrument, such as the development of cognitive and psychomotor skills, improved visuospatial skills, and bimanual dexterity, and better hand-eye coordination [15,16]. In a health care setting, a prospective cross-sectional study of 51 medical students evaluated the influence of previous musical experience on surgical skills in terms of speed and quality of performance. Students with previous musical experience performed better than nonmusicians [15]. Another study of 30 medical students evaluated the influence of previous musical experience on laparoscopic suturing tasks and found increased performance speed in musicians [17]. Furthermore, surgical and musical practice appear to share common cognitive skills such as accuracy of motor performance, integration of multimodal sensory and motor information, hand-eye coordination, spatial visualization, intense concentration, short reaction time, and efficient mental rotation [18].

The nonsurgical skills developed by playing a musical instrument appear to be similar to those developed by playing video games. These similar nonsurgical skills have been compared to assess their impact on surgical skills. A cohort study of 57 students using a simulator showed that subjects exposed to both video games and playing a musical instrument had higher psychomotor skills [18]. The influence of music, as opposed to video games, has been demonstrated in microsurgery [19]. However, some studies have shown no improvement in surgical or microsurgical performance in video game players and musicians [20,21].

Consequently, there is currently no consensus in the literature on the impact of these leisure activities on learning in medical education. Furthermore, there is no previous study of this issue in the context of dental education.

### Study Objectives

In this context, the aim of our study is to evaluate the impact of certain leisure activities, such as playing video games or playing a musical instrument, on the learning of technical skills by dental students. Using baseline measurements, we will observe whether initial skills differ between students who engage in one of these 2 leisure activities and those who do not. Subsequently, longitudinal monitoring will allow us to follow the development of students’ learning of these three skills, that are (1) mental rotation of an object, (2) precision and hand-eye coordination, and (3) speed of execution, according to their leisure activities.

## Methods

### Study Design and Setting

The purpose of this protocol is to establish a study that would be prospective, monocentric, controlled, longitudinal, and conducted at the Faculty of Dental Surgery in Nantes from September 2023 to April 2025.

### Study Population

The study population includes second-year surgical students who started their studies in September 2023 and have not yet started preclinical training with simulation-based placements. These students are followed until the end of their simulation training, that is, the end of the third year.

### Sample Size

The planned number of subjects included in the study would be 86, representing a full year’s intake at our faculty.

### Eligibility Criteria

Inclusion criteria are that the participants must be major dental surgery students enrolled in their second year at the Faculty of Dental Surgery of Nantes. Exclusion criteria are that the student is a minor, repeats a year in the second year, or does not agree to participate in the study. Inclusion or exclusion criteria will be reviewed by the investigator for each subject after completion of the preliminary questionnaire, that we created for this study, in the case report form (CRF). They must give verbal consent after being informed about the study by the investigators. The student must not have any particular physical or mental condition that prevents them from carrying out their practical training under the same conditions as other students. This information was collected on the basis of a previous medical diagnosis, according to the student’s statements.

Demographic characteristics were collected using the preliminary questionnaire completed by the participants at the start of the study. This allowed us to collect data such as gender, age, and leisure habits.

### Allocation

Based on the answers to the preliminary questionnaire, the students are divided into different groups according to their music or video game experience. Assignment of the subject to the “video game players” group if the student describes an hourly habit of playing action video games (first-person shooter or role-playing games) based on 0-60 minutes per week for occasional players, 61-300 minutes per week for moderate players, or more than 300 minutes per week for regular players. These values were used in a 2016 study to link the time spent playing video games to children’s mental health and cognitive and social skills [22].

Assignment of the subject to the “music players” group if the student describes an hourly habit of using a musical instrument based on 0-60 minutes per week for occasional players, 61-300 minutes per week for moderate players, or more than 300 minutes per week for regular players. These values are used to be consistent with those used to define a video game player profile. The methods used in other studies to define a music player were either too subjective or too complicated to implement. The different groups “video game players,” “music players,” and “nonplayers” can thus be constituted.

### Study Timeline

**Figure 1:**
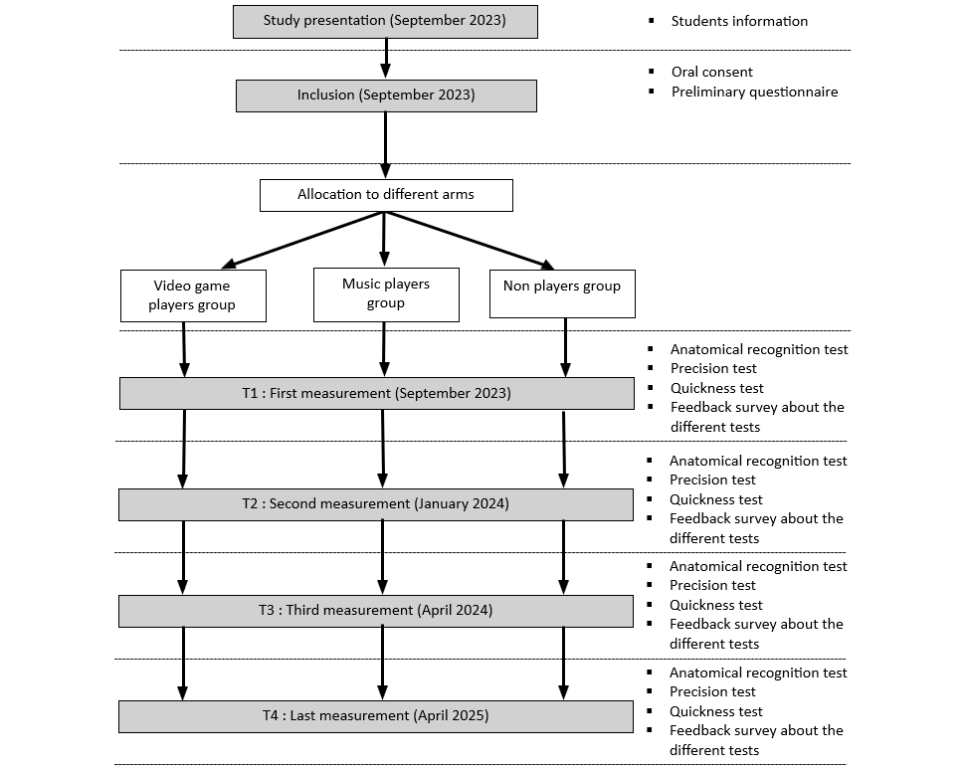
Flow Chart of the Study Procedure.

This study therefore consists of 4 phases of investigation: the first, which serves as a baseline evaluation, and the subsequent phases at 6 months, 1 year, and 2 years, which are useful for measuring the progress of the different groups during the preclinical phase of dental surgery studies, which in this case lasts 2 years. The timetable for the study is shown in Figure 1. It shows the various tests carried out during each measurement period. Each experimental session takes place on a single day. Each phase of the study takes place over the course of a day, in 4-time slots of 1 hour and 40 minutes each, with the total number of students divided into 4 groups of about 20.

### Outcomes

We aim to assess whether the initial level and development of preclinical skills over the 2 years of preclinical education show superior or faster progress among students identified as “music players” or “videogames players” compared with “nonplayers.” To achieve this, our study will focus on 3 main objectives to compare the progress of these different groups over time.

#### Objective 1

Objective 1 was to assess baseline measures and learning progress between the “players” group (video games or music or both) and “nonplayers” in their ability to mentally visualize 3D volumes. Comparative analysis of mean test scores will be conducted at all 4 planned stages.

**Figure 2:**
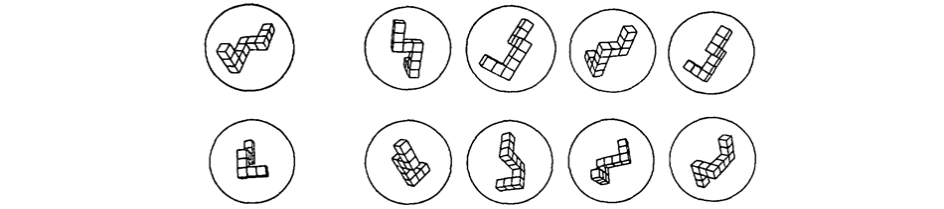
Sample items for the Vandenberg mental rotation test.

The evaluation criterion will refer to the score obtained from Vandenberg and Kuse’s mental rotation test [23], which was used in 2009 to measure the spatial representation skills of dental students [14] (Figure 2). The stimuli are 3D structures consisting of a line of 9 to 10 cubes angled at 3 points. Some of the presented items consist of identical stimuli that can be matched by rotation in the frontal or sagittal plane. The other items are pairs of completely different stimuli. Each item consists of 5 figures, a model placed at the left end of the line, and 4 structures to the right of the model, among which the subject must indicate those that resemble the model. There are always 2 correct answers for each item. The test consists of 20 items divided into 4 pages of 5 items each. For half of the items, the incorrect figures are rotations of the mirror image of the model, while the other half are rotations of 1 or 2 other structures. For high school and university students, the author recommends limiting the time to 6 minutes, that is 2 3 minutes [23]. A total of 2 points are awarded for each line with 2 correct choices, 1 point if only one of the drawings is selected and is correct, 0 points if 1 of the 2 choices is correct and the other is not, or if both are incorrect. This system eliminates the effect of random answers. We allow 3 minutes for each of the 2 sections of the test, with 10 items per section. The maximum possible score for this test is therefore 40. The purpose of these tests is to assess the student’s anatomical representation skills and how they develop over the year according to the student’s profile. At the end of this first test, the student fills out a feedback questionnaire on this anatomical recognition test.

#### Objective 2

Objective 2 was to assess baseline measures and learning progress between the “players” group (video games or music or both) and “nonplayers” in terms of precision of hand gestures using both right and left hands (eye-hand coordination). A comparative analysis of the average scores of the corresponding test will be conducted across all 4 phases.

**Figure 3:**
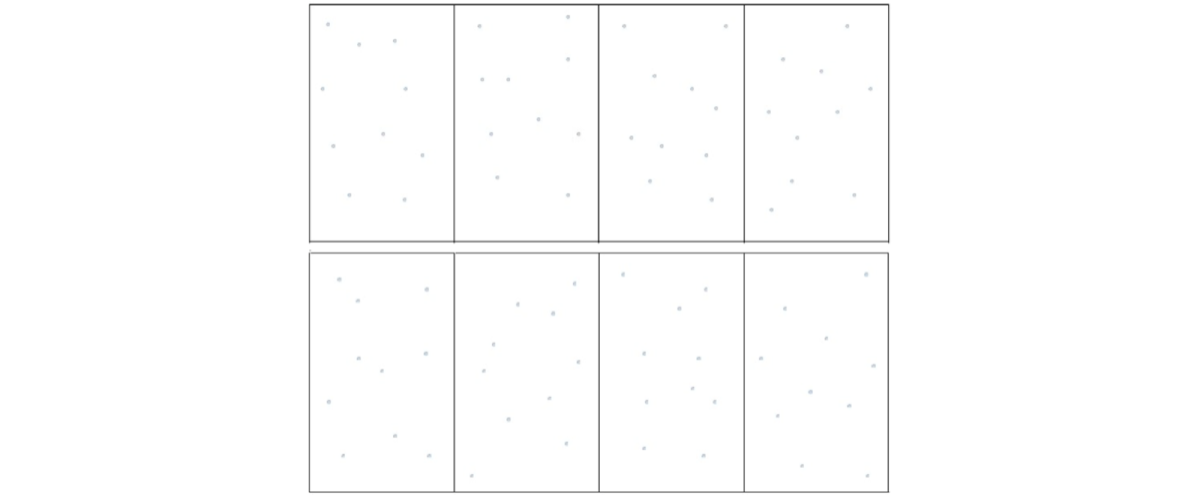
Modified Precision Manual Dexterity test.

The evaluation criterion will relate to the result obtained in the modified Precision Manual Dexterity (PMD) test as described by Bowers et al [24] (Figure 3). The test consists of penetrating 80 targets printed on a sheet of paper stretched over a soft surface as accurately as possible using a hand-held 15/100 steel endodontic hand file, with the right hand on the 4 rectangles on the right side of the sheet (ie, 40 targets) and with the left hand on the 4 rectangles on the left side of the sheet (ie, 40 targets). This allows us to test students with both hands (dominant or nondominant) [7,21]. The test sheet is divided into 8 target areas, each containing 10 randomly placed targets, the diameter of which has been increased to 1 mm, the initial PMD test using optical aids, and magnification 8 and 2.5. A scoring system is used to assign a total score to the test sheet: 3 points if the penetration point is completely in the target, 2 points if the penetration point is at least 50% on the target, 1 point if the penetration point is at least 50% off the target, 0 points if the penetration point is completely off the target. A prepunched sheet with a score of 240, the maximum for each hand, is used as a reference for scoring and compared with the player’s sheet using the backlight. The maximum total score is 120 points for the right hand and 120 points for the left hand. The accuracy of the subject’s gesture is the parameter evaluated. At the end of this second test, the subject fills out a feedback questionnaire on this hand-eye coordination test.

#### Objective 3

Objective 3 was to assess baseline measures and learning progress between the “players” group (video games or music or both) and “nonplayers” in terms of speed of gesture execution. A comparative analysis of the average scores of the corresponding test will be performed at each of the 4 stages.

**Figure 4:**
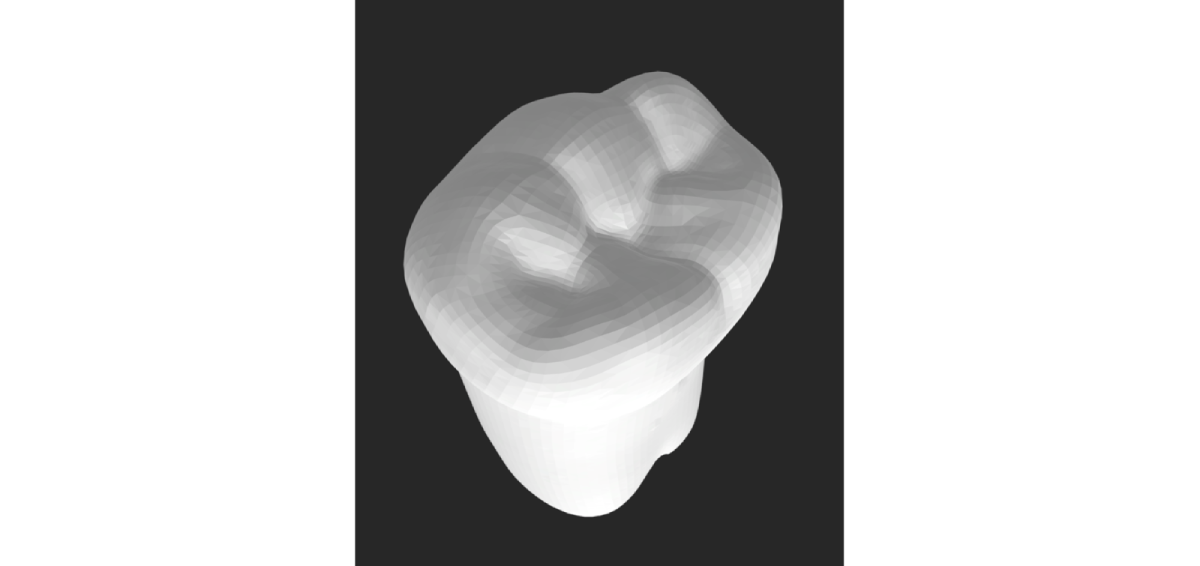
Stereolithography file of a 3D printed model of the permanent tooth.

The assessment criterion will relate to the time taken by the student to perform a complete pulpotomy on a mandibular permanent molar. The test is performed on practical models mounted on preclinical simulation mannequins or “phantoms.” A 3D printed model of the permanent tooth (right permanent mandibular first molar) in resin with a space simulating the pulp chamber is used to best simulate a real clinical situation (Figure 4). Just before performing the technical procedure, subjects are given appropriate instructions and success criteria. For each student, the investigators record the time (in minutes) required to perform the pulpotomy correctly. The parameter evaluated in this test is therefore the speed of performance, by comparing the average time taken by the groups during the recordings. At the end of this third test, the subject fills in a feedback questionnaire on this pulpotomy test.

### Data Collection

#### Identification of Participants

Investigators agree to keep the identity of research participants confidential by assigning them a code. This code will be used for all CRFs. It is the only information that can be used to identify the participant. Therefore, this study will be conducted in a single-blind fashion. Anonymization of participants in the CRF was carried out by the investigators at the time of enrolment.

Each student’s personalized CRF contains their answers to the anatomical recognition test, the time taken for the speed test, and their score on the accuracy test, for all 4 iterations.

#### Statistical Analysis Plan

A descriptive analysis of the sample will be performed using counts and percentages for categorical variables and means and SD for continuous variables. Missing data will not be imputed. Depending on the size of the samples of video game and music players, nonparametric statistical tests will be used to compare the mean scores on the 3 different tests at the 4 planned stages. A comparison of the mean scores on the feedback questionnaire for each test is made at each of the 4 stages. Each comparison will be made for the categories of “video game players” versus “video game nonplayers” and “music players” versus “music nonplayers” groups. A total of 2 researchers, JT and CG, will be single-blinded, meaning they will not know which group the students belong to, to enter data and analyze results. The data collected will be described as median, SD, and IQR. For nonparametric data, the statistical test appropriate to the size of the groups (Mann-Whitney *U* test) will be used to compare medians between groups. The significance level was set at 5%. The data collected are compared between the different groups. Qualitative data from the intervention will be entered into Microsoft Excel (version 16.86).

### Ethical Considerations

Our protocol was reviewed and approved by the Ethics, Deontology, and Scientific Integrity Committee of the University of Nantes, France (institutional review board approval number IORG0011023). All procedures performed in this study will be in accordance with the ethical standards of the Ethics, Deontology, and Scientific Integrity Committee of the University of Nantes and with the Declaration of Helsinki of 1964 and its subsequent amendments. Informed consent will be obtained from each student participating in the study.

## Results

All 86 nonrepeating second-year dental surgery students were enrolled in the study in September 2023. The first and second run of the study has already been completed and will result in an interim analysis. The next will take place in April 2024 and the last in April 2025. We plan to publish the baseline data on the influence of playing video games and a musical instrument separately for September 2024. A final publication including the influence of these 2 practices (jointly or separately) and the evolution of their impact over the 2 years of preclinical training is also planned for September 2025.

## Discussion

### Principal Findings

Playing video games and practicing some musical instruments seem to be promising ways to improve manual dexterity and cognitive skills. Our aim is to investigate the relevance of playing these activities in the context of learning dentistry. Specifically, we aim to determine whether regular video games or musical instrument practice has a positive impact on the baseline preclinical performance of novice dental students. In addition, our study aims to determine whether these “video game players” and “music players” students progress more quickly in their preclinical skills than their “nonplayers” peers.

### Public Health Impact

The acquisition of technical skills in dental students is critical to their future effectiveness as health care providers. Understanding these skills through innovative methods, such as video games and music engagement, can lead to better-prepared graduates who are proficient in performing complex dental procedures. This has a significant public health impact as it can lead to better oral health outcomes and overall health for the population.

### Existing Literature

Numerous studies have investigated various methods to enhance technical skills in medical and dental education. For example, the use of simulation-based training has been widely documented for its effectiveness in improving clinical skills. However, there is limited research on the impact of video games and music on technical skills acquisition. Some studies have suggested that video games can improve hand-eye coordination and cognitive skills, which are essential for dental procedures. Similarly, music has been found to enhance concentration and reduce anxiety, potentially leading to better performance in skill-based tasks.

### Rationale for Studying Dental Students

Dental students were chosen for this study because their training requires a high level of manual dexterity and precision. The preclinical phase is a critical period during which students develop foundational technical skills that they will use throughout their careers. By focusing on dental students, we aim to explore innovative methods to enhance these essential skills during this formative stage. In addition, dental students represent a homogeneous group in terms of educational background and training requirements, allowing for more controlled and consistent study conditions.

### Study Methodology

The decision to conduct a prospective, monocentric, controlled, longitudinal study was made to ensure rigorous and reliable results. A prospective design allows us to observe the effects of video games and music engagement over time, while a controlled setup helps in comparing the intervention group with a control group to determine the specific impact of the interventions. A monocentric study conducted at the Faculty of Dental Surgery in Nantes provides a consistent environment and resources, facilitating detailed monitoring and data collection over the study period from September 2023 to April 2025.

### Strengths

Our study has a few strengths, including its innovative aspect, as it does not appear that similar work has been carried out in dental education. In addition, it is a longitudinal study, which should allow us to monitor the results obtained at the beginning and follow their evolution in the different groups. In addition, the participants started the study before starting any dental surgery training, whether theoretical or practical, to limit any possible bias. The tests used to assess the participants had been tried and tested in previous studies.

### Limitations

As the study is monocentric, it might be interesting to distribute the preliminary questionnaire to other French faculties to confirm that our sample is representative of our target population.

### Plan of Dissemination

We will publish all results in peer-reviewed international journals indexed in Web of Science or Scopus databases and present them at national and international conferences. Depending on the interest and importance of the results and the relationship between music and video game playing and preclinical skills, a decision may be made to disseminate the results in the form of one or more articles.

### Conclusion

In France, dental students present very heterogeneous profiles at the beginning of their university studies to become dental surgeons. Coming from different recruitment systems and scientific or nonscientific backgrounds, their knowledge and technical skills seem to be uneven. The results of our research could potentially make it possible to tailor students’ practical workload according to their needs of formation, by focusing on their leisure activities, such as video games and music. This knowledge could facilitate personalized support for students who are not naturally talented and require more diligent training. Finally, this work could argue for the integration of video games and music into medical education, based on evidence of their benefits in a learning context. We are currently in a situation where the number of students continues to grow while the university infrastructure does not allow us to accommodate any more. Identifying learner profiles and adapting the simulation time required for each would allow us to optimize the training of our students and manage the availability of facilities as effectively as possible.

## Abbreviations

CRF: case report form
PMD: Precision Manual Dexterity

## Data Availability

The data sets generated during and/or analyzed during this study are available from the corresponding author on reasonable request.
